# Induced parental care in a poison frog: a tadpole cross-fostering experiment

**DOI:** 10.1242/jeb.165126

**Published:** 2017-11-01

**Authors:** Andrius Pašukonis, Kristina Barbara Beck, Marie-Therese Fischer, Steffen Weinlein, Susanne Stückler, Eva Ringler

**Affiliations:** 1 FAS Center for Systems Biology, Harvard University, Cambridge, MA 02138, USA; 2 Department of Cognitive Biology, University of Vienna, 1090 Vienna, Austria; 3 Department of Behavioural Ecology and Evolutionary Genetics, Max Planck Institute for Ornithology, 82319 Seewiesen, Germany; 4 Department of Integrative Zoology, University of Vienna, 1090 Vienna, Austria; 5 Messerli Research Institute, University of Veterinary Medicine Vienna, Medical University of Vienna, University of Vienna, 1210 Vienna, Austria

**Keywords:** Parental behaviour, Behavioural trigger, Flexibility, Spatial cognition, Tadpole transport, Telemetry

## Abstract

Understanding the external stimuli and natural contexts that elicit complex behaviours, such as parental care, is key in linking behavioural mechanisms to their real-life function. Poison frogs provide obligate parental care by shuttling their tadpoles from terrestrial clutches to aquatic nurseries, but little is known about the proximate mechanisms that control these behaviours. In this study, we used *Allobates femoralis*, a poison frog with predominantly male parental care, to investigate whether tadpole transport can be induced in both sexes by transferring unrelated tadpoles to the backs of adults in the field. Specifically, we asked whether the presence of tadpoles on an adult's back can override the decision-making rules preceding tadpole pick-up and induce the recall of spatial memory necessary for finding tadpole deposition sites. We used telemetry to facilitate accurate tracking of individual frogs and spatial analysis to compare movement trajectories. All tested individuals transported their foster-tadpoles to water pools outside their home area. Contrary to our expectation, we found no sex difference in the likelihood to transport or in the spatial accuracy of finding tadpole deposition sites. We reveal that a stereotypical cascade of parental behaviours that naturally involves sex-specific offspring recognition strategies and the use of spatial memory can be manipulated by experimental placement of unrelated tadpoles on adult frogs. As individuals remained inside their home area when only the jelly from tadpole-containing clutches was brushed on the back, we speculate that tactile rather than chemical stimuli trigger these parental behaviours.

## Abstract (precis)

**Summary:** Placement of unrelated tadpoles on adult poison frogs triggers a cascade of parental behaviours involving tadpole transport and spatial memory use in both sexes, despite the asymmetric parental sex roles.

## INTRODUCTION

Studying the external stimuli and contexts that induce and modulate complex behaviours, such as parental care, is key to linking the proximate mechanisms to the function of behaviour (Brown, 1985; Taborsky et al., 2015). Understanding the constraints imposed by these proximate mechanisms on behavioural flexibility can also provide insight into the evolution of traits associated with parental care. For example, the predisposition of many birds to respond to orange gape and loud begging calls with food provisioning explains, at least in part, the prevalence of brood parasites among altricial birds (Kilner et al., 1999; Redondo, 1993). Despite the diversity of vertebrate parental strategies (reviewed in Balshine, 2012), it has been suggested that many neuroendocrine mechanisms might be conserved across taxonomically distant groups (Brown, 1985; Dulac et al., 2014). Cross-fostering of young has been used to study the neural and hormonal basis of parental care, offspring recognition, adaptive value of parental investment and other associated traits in many vertebrates (e.g. Buntin, 1996; Dewsbury, 1985; Francis et al., 1999). Amphibians show a variety of parental behaviours, including egg guarding, nest making, larval transport and provisioning (Crump, 1996; Wells, 2007), but the proximate mechanisms of amphibian parental care remain mostly unknown and standard methods such as cross-fostering are rarely applied (but see Ringler et al., 2016a; Stynoski, 2009).

Poison frogs (Dendrobatoidea) are a well-studied group of Neotropical frogs with complex life histories that involve parental care, including egg attendance and larval transport from terrestrial clutches to aquatic deposition sites (Grant et al., 2006; Weygoldt, 1987). The adaptive value and the evolution of parental care in this group has attracted a considerable amount of research (reviewed in Summers and Tumulty, 2013), but the proximate mechanisms of these behaviours remain poorly understood (Roland and O'Connell, 2015). Several recent studies on the poison frog *Allobates femoralis* have revealed a surprising degree of flexibility in the parental care strategies of this species. In *A. femoralis* tadpole transport is primarily performed by males (Ringler et al., 2013). While males indiscriminately transport all tadpoles encountered inside their territory (Ringler et al., 2016a), males become cannibalistic when establishing a new territory (Ringler et al., 2017), thus adjusting their parental responses towards unrelated clutches according to their territorial status. Females, in turn, show compensatory parental care when the father disappears (Ringler et al., 2015a), and will only transport tadpoles from the exact location of their own clutch. Neither sex appears to use direct cues for offspring recognition (Ringler et al., 2016a).

**Fig. 1.**
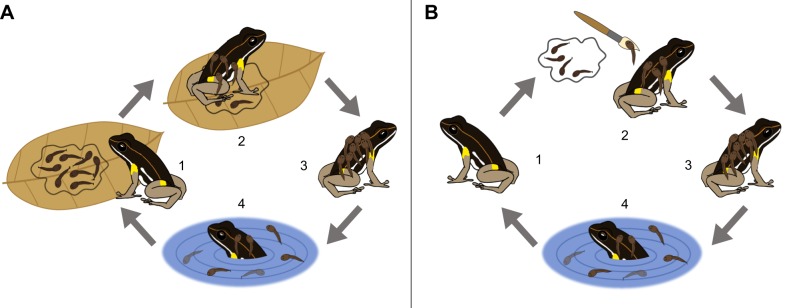
**Schematic representations of** **the behavioural cascade involved in tadpole transport.** (A) Natural tadpole transport. The numbered stages represent: (1) clutch recognition and homing; (2) tadpole pick-up; (3) orientation and tadpole transport; (4) pool inspection and tadpole deposition. (B) Induced tadpole transport. The numbered stages represent: (1) adult in home area and homing; (2) tadpole transfer from an unrelated clutch to the back of a frog; (3) orientation and tadpole transport; (4) pool inspection and tadpole deposition.

Tadpole transport in *A. femoralis* follows a sequence of stereotypical behaviours (Fig. 1A, Movie 1). First, to pick up the tadpoles, the parent lowers its body posture and rotates in the clutch, waiting for the tadpoles to wriggle onto its back. The tadpole carrier will then transport the offspring to, and sometimes distribute them between, small terrestrial pools, tens to hundreds of metres away from the territory (Beck et al., 2017; Erich et al., 2015; Ringler et al., 2013). Once at the pool, the parent appears to wait and inspect the site before finally submerging itself in the water and allowing the tadpoles to swim off (A.P., K.B.B., M.-T.F., S.W., S.S. and E.R., personal observation). After the deposition, the parent returns to the home territory via a direct path (Beck et al., 2017). Recent tracking studies have revealed that *A. femoralis* relies on large-scale spatial memory for finding the pools and homing (Beck et al., 2017; Pašukonis et al., 2014a,b, 2016). Further, it has been proposed that some strategic planning of where to go and how many tadpoles to transport is involved (Ringler et al., 2013). Together, these finding suggest that the stereotypical action patterns involved in parental care are controlled by a fairly flexible decision-making process and extensive use of spatial memory. Understanding the stimuli that trigger and control such a behavioural cascade would constitute a crucial step in understanding the proximate mechanisms involved. In turn, understanding the proximate mechanisms of parental care in poison frogs would provide key insights into the evolution of vertebrate parental care as well as amphibian cognition.

In this study, we used telemetry and an array of artificial deposition sites to test whether tadpole transport can be triggered in male and female *A. femoralis* by experimentally transferring tadpoles to the backs of unrelated adults in the field. Several decision-making rules involved in *A. femoralis* parental care have been demonstrated or hypothesized in recent years, such as the use of spatial cues for offspring recognition, context-dependent decision to transport, and context-dependent number of tadpoles transported (Ringler et al., 2013, 2015a, 2016a, 2017). We were interested in whether the presence of tadpoles on the back of an adult can override these decision-making rules naturally preceding the tadpole pick-up and induce the recall of spatial memory necessary for finding the tadpole deposition sites. Because females are more selective in initiating parental care in this species, we expected females to be less likely to transport cross-fostered tadpoles. In addition, we expected males to be faster and more accurate in finding suitable tadpole deposition sites because of their predominant role in tadpole transport.

## MATERIALS AND METHODS

### Study area and species

The study was carried out from January to March 2016 and 2017 in an experimental population established on a 5 ha river island (Ringler et al., 2015b) near the Camp Pararé field site at the CNRS Nouragues Ecological Research Station in the Nature Reserve Les Nouragues, French Guiana (http://www.nouragues.cnrs.fr). The frogs in the study area rely primarily on an array of 13 artificial plastic pools (volume ∼10 l, inter-pool distance ∼20 m) for tadpole deposition.

*Allobates femoralis* (Boulenger 1884) is a small diurnal frog common throughout the Amazon basin and the Guiana Shield (Amezquita et al., 2009). During the rainy season, males establish acoustically advertised territories on the forest floor where courtship and oviposition occur (Ringler et al., 2009). After 12–20 days of larval development, frogs transport 1–25 tadpoles and deposit them as far as 185 m from their territory in a variety of small terrestrial water bodies (Beck et al., 2017; Ringler et al., 2013). *Allobates femoralis*’ mating system is polygynandrous, most mature individuals reproduce continuously throughout the rainy season, and males have been observed caring for up to five clutches simultaneously (Ursprung et al., 2011).

### Frog sampling

At the onset of the experiment, we performed a baseline sampling of all females and males in the study area. We caught the frogs with transparent plastic bags, photographed them for individual identification by their unique ventral coloration pattern (Ringler et al., 2015b), and marked their position on a detailed GIS background map (Ringler et al., 2016b) using a tablet (WinTab 8, Odys) with the mobile GIS software ArcPad 10.0 (ESRI). We sexed the individuals by the presence (male) or absence (female) of vocal sacs. Male territorial behaviour (calling, aggression, courtship) and repeated recaptures allowed identification of male territories.

To obtain tadpoles for the experiments, we searched through the leaf-litter around the males' calling sites. In *A. femoralis*, clutches are typically laid on dry leaves within the male's territory. We marked the locations and followed the development of all detected clutches. To ensure that tadpoles used for experiments were morphologically ready to be transported, we only used hatched tadpoles in the appropriate developmental stage [Gosner stage 24–25 (Gosner, 1960); usually 12–16 days after oviposition; Fig. S1]. When not transported to the water, *A. femoralis* tadpoles can remain in the egg-jelly for many days after hatching (S.W., unpublished data).

### Experimental procedure

All experiments were started in the morning between 07:30 h and 10:30 h, which corresponds to the daytime period when *A. femoralis* usually initiate tadpole transport (Ringler et al., 2013). We captured the frogs in what we assumed were their home areas, fitted them with a transponder for tracking and subjected them to one of two experimental treatments: (1) tadpole cross-fostering (tadpole group hereafter) or (2) egg-jelly swabbing (jelly group hereafter). The second treatment (jelly) was conducted in the second field season as an addition to the first treatment (tadpoles). Each experimental group included 10 males and 10 females and we did not use the same individuals between the two field seasons.

### Tadpole group

**Fig. 2.**
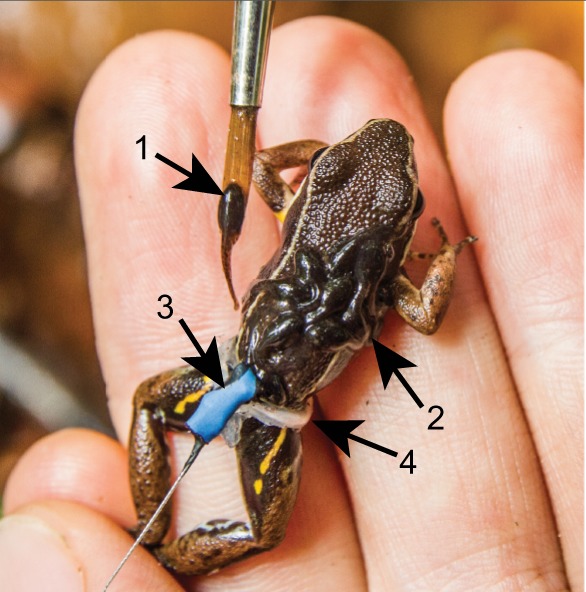
**Photograph of a captured frog wearing the tracking transponder during the tadpole transfer procedure.** The numbers and arrows indicate: (1) a live tadpole being transferred with a fine-tipped paintbrush; (2) tadpoles already transferred on the back of the frog; (3) a sealed diode with antenna used for tracking; (4) the silicone waistband holding the tag.

To test whether tadpole transport can be induced, we forcibly placed tadpoles on frogs' backs. We first carefully removed the tadpoles from the jelly onto a moist leaf using a fine-tipped paintbrush. We then transferred the tadpoles one-by-one from the leaf to the back of an adult while holding the frog immobilized by the hindlegs (Fig. 2; Movie 2). We transferred 8–12 tadpoles, which corresponds to the average number of tadpoles transported naturally. We waited for a few minutes until the tadpoles settled on the back, then released the frogs as gently as possible to prevent the larvae from falling off. Occasionally, the frog lost a few tadpoles with a sudden escape movement during release. We never observed the frog losing additional tadpoles after the initial release. We continuously observed the frog for 5–15 min immediately after release, during which time most frogs remained immobile. During this period, we often observed the tadpoles wiggling on the frog and sometimes repositioning themselves on its back. To minimize the chances of testing a male with his own tadpoles, we only used tadpoles collected outside the respective male's territory and its immediate neighbourhood (at least 30 m from the tested male). To minimize the chances of testing a female with her own tadpoles, we used tadpoles collected at least 50 m away from the capture site of each tested female, as females preferentially mate with males within an approximately 20 m radius (Ursprung et al., 2011).

### Jelly group

To control for the effects of handling and tagging and to test whether tadpole transport can be induced by chemical cues present in the egg-jelly, we did a follow-up experiment in 2017. We dipped the paintbrush into a clutch containing at least eight ready-for-transport tadpoles and gently brushed the egg-jelly on the back of the frog. The procedure was repeated eight times and the frogs were handled identically to the tadpole group.

### Tracking

We used the harmonic direction-finding telemetry technique to track individuals and followed a protocol that has been successfully used in *A. femoralis* in several contexts including tadpole transport (Beck et al., 2017; Pašukonis et al., 2014a,b). The system consists of a directional transceiver and a passive reflector (i.e. transponder) attached to the animal. The tag together with the attachment comprised less than 5% of the frog's body mass (frog mass _ _∼2 g, tag mass <0.1 g). We used a commercially available transceiver (R8, RECCO^®^ Rescue System, Lidingö, Sweden) for tracking. Following release, all frogs were located every 15–30 min during their daylight activity hours (∼07:30 h to 18:30 h). In each case, we carefully approached the location of the signal and attempted to detect the frog visually. In cases of poor visibility or if an individual was hiding, we narrowed the signal to less than 1 m. Every position fix was recorded on the background GIS map as described above. Whenever the frog was visible, we also recorded the number of tadpoles present and the current behaviour (e.g. moving, hiding, depositing tadpoles). In 2016 (tadpole group), we followed the frogs until all tadpoles were deposited. Most frogs completed tadpole transport and deposition within 1 day (mean±s.d. duration 4.47±2.48 h), but two individuals were tracked for 2 consecutive days (Table S1). All males were untagged immediately after tadpole deposition and were recaptured back in their territory over the following days. As females do not show conspicuous behaviour such as calling or territorial aggression, it was not always possible to confirm female homing by recapture. Thus, we tracked three females that had moved the longest distance after deposition to confirm that females also return to their home areas after tadpole transport. All three females tracked showed homing behaviour and moved back to their original home areas after tadpole deposition. In 2017 (jelly group), the frogs were tracked for 1 day between 6.7 h and 9.4 h (mean duration 7.92 h) and untagged in the evening between 17:30 h and 18:45 h.

### Movement analysis

Visualization and extraction of coordinates were done in the GIS software ArcGIS™ 10 (ESRI). To describe the spatio-temporal characteristics of frog movement to the pools, we quantified the latency to move, the speed of movement and the distance travelled to the first deposition site, and the total duration until all tadpoles were deposited. The latency to move was defined as the time taken to leave a 5 m radius area from the capture/release site. This cut-off was chosen based on the distance to the closest deposition site across all frogs and it allowed us to separate various local movements around the release site from the directional movement towards the deposition site. As *A. femoralis* do not move at night, for the two frogs that were tracked overnight, we subtracted 12 h for the calculation of movement speed. We compared the total duration of tadpole transport between males and females with and without subtracting the 12 h.

To estimate orientation accuracy, we calculated a straightness coefficient (SC), defined as the ratio between the straight-line distance and the actual path distance between the starting point and the first deposition site (such that SC=1 would indicate a perfectly straight path). SC is a simple but robust estimator of the efficiency of a goal-oriented path (Benhamou, 2004). For the frogs that did not visit a deposition site, we calculated the latency to move, the total path length, the distance to the farthest point moved from the capture sites, and the movement speed over the entire tracking period. We performed a Mann–Whitney *U*-test for sex differences in temporal and spatial parameters. We also used the Mann–Whitney *U*-test to compare the speed of movement and SC of induced male tadpole transport with natural male tadpole transport (see Beck et al., 2017). No tracking data on natural female tadpole carriers are currently available. For visual representation, we plotted pool direction-normalized trajectories derived from the linear interpolations of consecutive positions. All analyses were performed in GIS software ArcGIS™10 (ESRI) and R statistical software (http://www.R-project.org/).

### Ethical statement

Our study was approved by the Animal Welfare Board of the University of Vienna (approval number: 2016-003) and by the scientific committee of the research station where fieldwork was conducted. All necessary permits were provided by the local authorities (DEAL: ARRETE no. 2011-44/DEAL/SMNBSP/BSP). All sampling was conducted in strict accordance with current French and EU law and followed the ‘Guidelines for use of live amphibians and reptiles in the field and laboratory research’ by the Herpetological Animal Care and Use Committee (HACC) of the American Society of Ichthyologists and Herpetologists.

## RESULTS

**Fig. 3.**
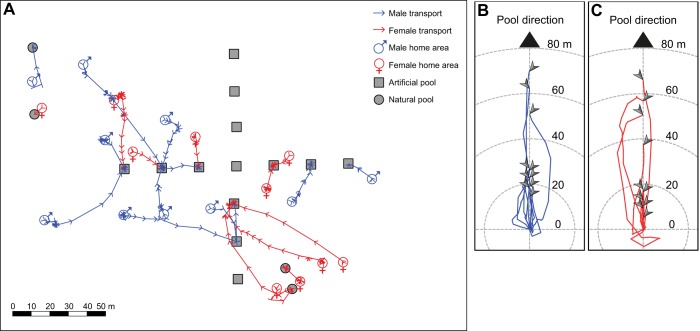
**Study area map and frog trajectories, showing the induced movements.** (A) Schematic representation of the study area and the movement patterns of tested frogs during induced tadpole transport from their home areas to aquatic deposition sites. Grey squares represent the artificial pool array; grey circles represent natural deposition sites visited by tested frogs. Symbols indicate female and male home areas. Red and blue lines represent interpolated female and male movement trajectories. Arrows on the lines mark every position recorded during tracking and indicate the direction of movement. (B,C) Tadpole transport trajectories of (B) males and (C) females normalized for and shown until the first deposition site. Each line corresponds to a single individual. The grey arrows indicate the last point of each trajectory, i.e. the deposition site.

All 20 individuals from the tadpole group transported and deposited the cross-fostered larvae. Seventeen frogs deposited in artificial pools and three frogs used natural pools (Fig. 3A). Two frogs (males) distributed tadpoles between two pools and one frog (female) visited a dried-out deposition site before moving to an artificial pool. From the capture site to the first deposition site, frogs moved on average 36.5 m (mean±s.d. path_female_=35±31 m, path_male_=38±20 m) at an average speed of 10 m h^−1^ (speed_female_=8.5±6 m h^−1^, speed_male_=11.7±6.7 m h^−1^). The average latency to leave the 5 m-radius area around the capture site was 1.92 h (latency_female_=2.02±1.43 h, latency_male_=1.83±1.59 h) and the total duration until deposition was on average 6.47 h (duration_female_=8±9.05 h, duration_male_=4.49±2.26 h). The average orientation accuracy (SC) was 0.87 (SC_female_=0.89±0.14, SC_male_=0.85±0.11). We did not find any significant differences between the sexes in any temporal and spatial parameters measured (Fig. 3B,C; Mann–Whitney *U*-test: *U*_latency_=44.5, *P*=0.7; *U*_full_ _duration_=48, *P*=0.9; *U*_daytime_ _duration_ =54.5, *P*=0.8; *U*_speed_=33, *P*=0.2; *U*_SC_=65, *P*=0.3). We also found no significant difference between movement speed and orientation accuracy of males during induced and natural tadpole transport (Mann–Whitney *U*-test: *U*_speed_=54, *P*=0.8; *U*_SC_=44.5, *P*=0.7).

Only one frog (male) out of 20 moved to a pool after being swabbed with jelly from tadpole-containing clutches. During the entire tracking period, jelly group frogs moved on average 8.16±6.45 m (mean±s.d.) at an average speed of 1.1±0.9 m h^−1^ and only three frogs (two males and one female) left the 5 m-radius area around the capture sites.

## DISCUSSION

We successfully triggered tadpole transport and deposition in all tested frogs by transferring unrelated tadpoles onto their backs. In contrast, all frogs except one subjected to the same handling procedure and brushed with jelly from tadpole-containing clutches remained in their home areas. Even though *A. femoralis* females rarely and selectively transport tadpoles, both sexes were equally likely to transport cross-fostered tadpoles. We also found no difference in the accuracy or speed of finding deposition sites between males and females.

Our findings show that the decision-making processes and motor actions involved in tadpole pick-up do not need to precede successful tadpole transport (Fig. 1B). They also confirm a recent finding that *A. femoralis* do not use direct cues for offspring recognition (Ringler et al., 2016a), similar to results for egg-feeding behaviour in the strawberry-poison frog, *Oophaga pumilio* (Stynoski, 2009). Our ability to experimentally bypass tadpole pick-up is especially surprising for females, which are very selective in terms of the context in which they naturally pick up and transport tadpoles (Ringler et al., 2015a) and the precise spatial cues for clutch recognition (Ringler et al., 2016a). It suggests that even though males and females show diverging offspring recognition strategies, the mechanisms underlying tadpole transport are likely to be shared between the sexes. In addition, the similar spatio-temporal patterns of tadpole transport between the sexes suggest shared underlying spatio-cognitive capabilities. While parental care in poison frogs is typically classified based on species- and sex-specific parental roles, anecdotal observations and some experimental data suggest that both the presumed species- and sex-specific parental roles may be more flexible than often assumed (Myers and Daly, 1983; Ringler et al., 2015a; Souza et al., 2017; Tumulty et al., 2014; Vacher et al., 2017; Wells, 2007, table 11.3, pp. 524–526). We speculate that the mechanisms underlying poison frog parental care are not species- or sex-specific and therefore allow strong plasticity through ontogeny as well as flexibility in adults.

Our study provides limited information about the exact cues that trigger tadpole transport, but we speculate that tactile stimuli may play a predominant role. Dorsal swabbing with jelly from clutches that contained ready-for-transport tadpoles did not trigger the parental behaviour, suggesting that chemical cues passively released by tadpoles are not sufficient as a trigger. Tadpoles wriggling up and onto the back of a parent could provide a variety of tactile cues to initiate tadpole transport. Tadpoles of several tropical anurans, including dendrobatid frogs, are known to use tactile and vibrational cues for parent–offspring communication in the context of begging behaviour (Jungfer, 1996; Jungfer and Weygoldt, 1999; Kam and Yang, 2002; Stynoski and Noble, 2012; Yoshioka et al., 2016). The tactile cues could work in combination with chemical cues actively released by the tadpoles once on the back of the parent, which might in part explain the delay in moving following tadpole transfer. The mechanisms by which tadpoles identify a suitable carrier and adhere to their back are not well understood, but are also likely to involve mechanical and chemical interactions (Grant et al., 2006; Lüddecke, 1999; Myers and Daly, 1980). Interestingly, different poison frog species vary in how strongly the tadpoles are attached to the back of the carrier (Myers and Daly, 1980; A.P., personal observation). Identifying these attachment mechanisms might provide crucial insight into the cues that trigger tadpole transport in poison frogs.

Even though tadpole transport was triggered in all tested individuals, there was a marked and highly variable latency between tadpole transfer and the initiation of movement towards a deposition site. Several factors such as the nature of the trigger or the spatio-cognitive processes preceding long-distance movement may influence this latency, but we speculate that the motivational/hormonal state of the individual is likely to be the key element. Complex cycles of neuroendocrine parental care regulation in mammals, birds and fish are often preceded by reproductive triggers such as mating (Brown, 1985; Dulac et al., 2014). In *A. femoralis*, females can produce clutches on average every 8 days and males commonly have several clutches simultaneously inside their territory (Ursprung et al., 2011). As a result, mating and parenting generally overlap, but the exact reproductive state and thus the parental motivation most likely vary between individuals. Our results suggest that reproductively active adults are indeed in general readiness for tadpole transport and the variable latency to initiate transport might reflect the exact reproductive state of each individual. Interestingly, male parental care has been shown to correlate negatively with territoriality and aggressiveness via steroid hormone levels in several vertebrates (Ros et al., 2004; Wingfield et al., 1990), including one frog species (Townsend and Moger, 1987). *Allobates femoralis* males appear to be an exception to this pattern as they maintain high levels of aggression and territoriality while being in a state of parental readiness. Neuroendocrine control of parental care in poison frogs has recently attracted some attention (Roland and O'Connell, 2015; Schulte and Summers, 2017), but to date the mechanisms of parental care in amphibians remain a virtually unexplored field.

To sum up, our results echo the findings of classical ethological studies on parental spatial behaviour of digger wasps (*Ammophila* spp.; Baerends, 1941) and beewolves (*Philanthus* spp.; Tinbergen, 1932) in revealing how a single trigger can induce a stereotypical behavioural cascade of fixed-action patterns. Key components of such a sequence are usually controlled and modulated by memory. In *A. femoralis*, the presence of tadpoles on the back induces a stereotypical sequence of orientation, fast directional movement, pool inspection, tadpole deposition and homing – behaviours dependent on the recall of long-term spatial memory (Beck et al., 2017; Pašukonis et al., 2014a, 2016). The exact cues that trigger tadpole transport remain to be studied, but we speculate that tactile stimuli play a predominant role. We believe that our findings provide key behavioural data from the field and an experimental approach for future studies on the neuroendocrine basis of parental behaviour and the associated cognitive processes.

## Supplementary Material

Supplementary information
