# Supplementary material for: Induced parental care in a poison frog: a tadpole cross-fostering experiment
Source: J Exp Biol. 2017 Nov 1;220(21):3949–54. doi: 10.1242/jeb.165126 (PMC5702076; doi:10.1242/jeb.165126)
Supplement: Supplementary information [file jexbio-220-165126-s1.pdf]

Table S1. All measured and calculated variables per individual for the tadpole group frogs. Each row corresponds to a different individual. Females indicated by “f”, males indicated by “m”. See materials and methods for detailed explanation.

ID	Tadpoles added	Duration (h)	Speed (m/h)	Total path (m)	Path to 1st pool (m)	Straight distance (m)	SC	Latency to move (h)	Deposition site
f1	11	24.57	3.79	46.59	46.59	37.30	0.80	0.35	pool
f2	8	0.38	13.28	5.09	5.09	5.09	1.00	0.38	palm bract
f3	11	3.38	5.31	16.81	16.81	16.49	0.98	1.67	pool
f4	8	1.68	7.22	10.22	10.22	9.38	0.92	0.50	pool
f5	9	1.67	10.74	17.91	17.91	17.70	0.99	0.67	pool
f6	8	5.33	2.86	13.36	13.36	12.96	0.97	4.33	pool
f7	8	24.5	5.99	68.93	68.93	56.50	0.82	3.50	pool
f8	8	4.00	22.22	66.65	66.65	66.02	0.99	1.00	dry burrow then pool
f9	8	9.00	10.23	92.04	92.04	50.51	0.55	1.00	pool
f10	8	5.50	3.12	11.95	11.95	10.25	0.86	3.50	pool
m1	8	6.50	5.74	33.50	33.50	25.78	0.77	0.83	pool
m2	8	6.77	8.66	58.61	58.61	50.97	0.87	1.13	pool
m3	12	1.62	27.61	28.07	28.07	17.92	0.64	0.23	fallen tree
m4	8	4.17	9.79	24.47	24.47	22.32	0.91	1.00	pool
m5	8	6.75	8.64	49.11	28.80	26.92	0.93	0.83	2 pools
m6	11	1.67	11.18	14.91	14.91	14.66	0.98	0.67	pool
m7	12	5.50	5.74	25.84	25.84	18.62	0.72	4.50	pool
m8	8	6.70	11.17	72.24	72.24	69.47	0.96	0.35	pool
m9	8	7.25	9.34	67.70	67.70	62.11	0.92	4.35	pool
m10	10	2.50	18.88	47.19	26.75	22.45	0.84	0.75	2 pools

Journal of Experimental Biology • Supplementary information

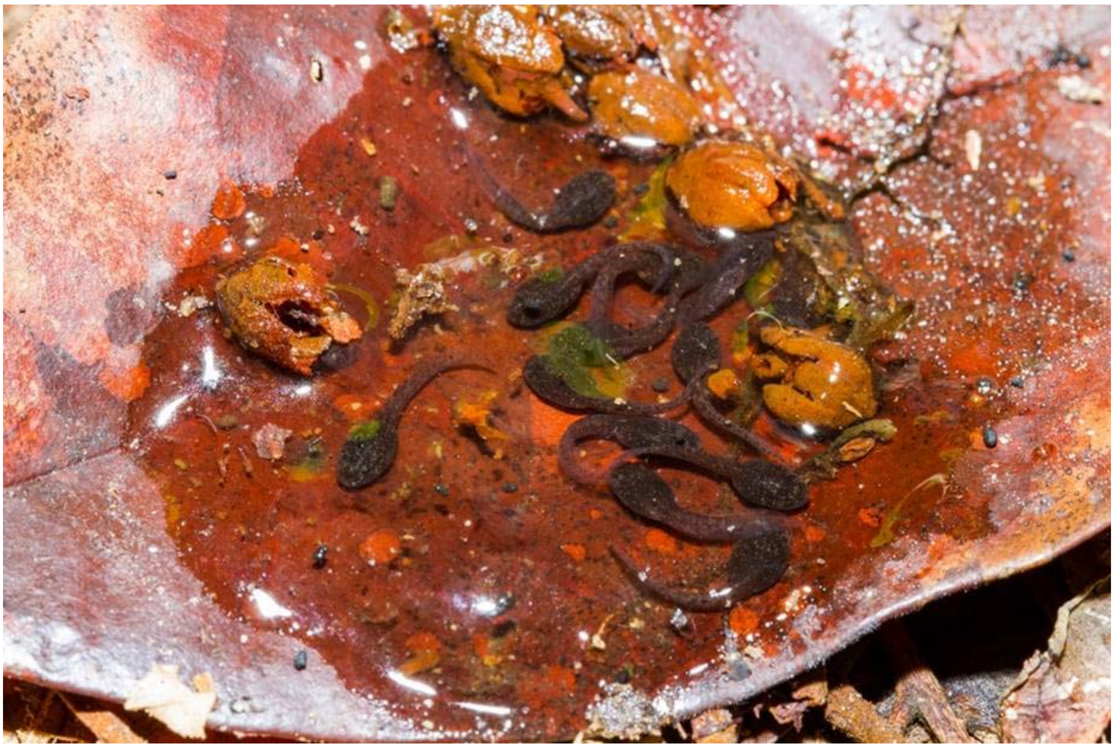

Figure S1. A photograph showing *A. femoralis* tadpoles hatched inside egg-jelly before being transported by an adult to the water. Tadpoles of similar developmental stage were used for inducing parental behavior.

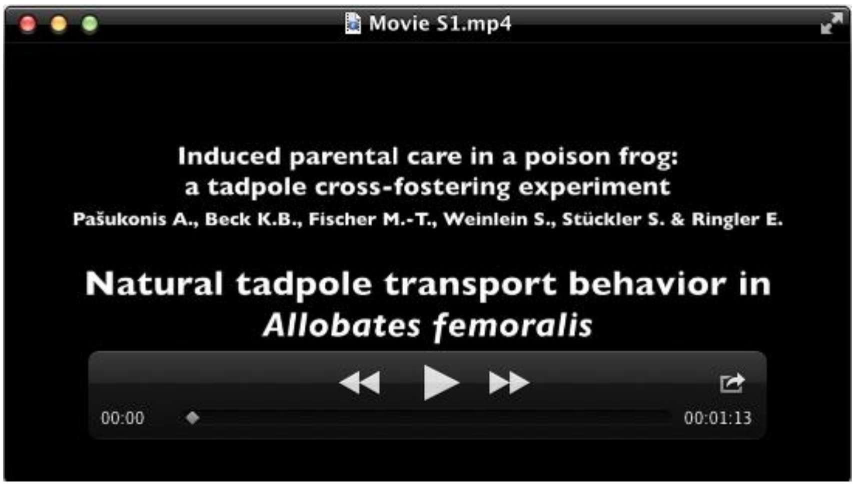

Movie 1

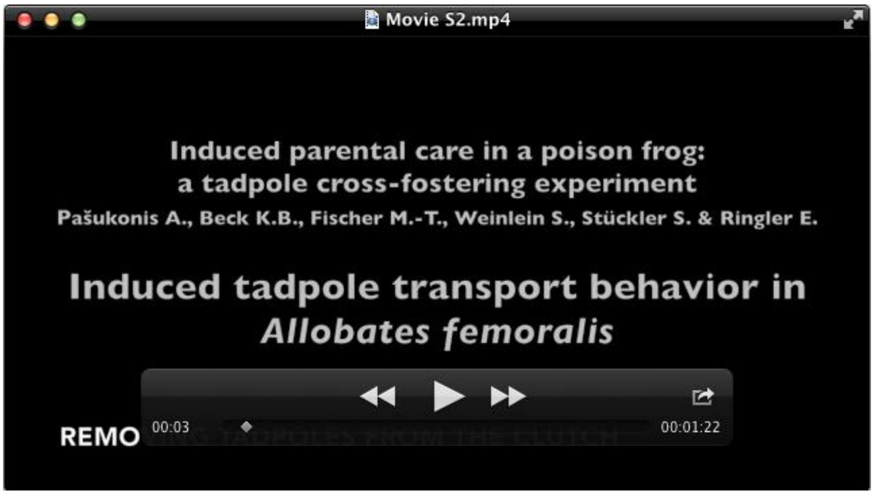

Movie 2

Journal of Experimental Biology • Supplementary information
